# Photosynthesis Inhibiting Effects of Pesticides on Sweet Pepper Leaves

**DOI:** 10.3390/insects11020069

**Published:** 2020-01-21

**Authors:** Miguel Giménez–Moolhuyzen, Jan van der Blom, Pilar Lorenzo–Mínguez, Tomás Cabello, Eduardo Crisol–Martínez

**Affiliations:** 1 Department of Economy and Business, University of Almeria, C\Universidad de Almeria, s/n, 04120 La Cañada, Almeria, Spain; miguel.gimenez@ual.es; 2 Department of Crop Production Techniques, COEXPHAL (Association of Vegetable and Fruit Growers of Almeria), C\Esteban Murillo, 3, 04746 La Mojonera, Almeria, Spain; jvdblom@coexphal.es; 3 Centro de Investigación en Agrosistemas Intensivos Mediterráneos y Biotecnología Agroalimentaria (CIAMBITAL), Agrifood Campus of International Excellence (CEIA3), University of Almeria, C\Universidad de Almeria, s/n, 04120 La Cañada, Almeria, Spain; tcabello@ual.es; 4 IFAPA, Centro La Mojonera, 04745 La Mojonera, Almeria, Spain; pilar.lorenzo@juntadeandalucia.es; 5 EcoLaVerna Integral Restoration Ecology, Bridestown, Kildinan T56CD39, Co. Cork, Ireland

**Keywords:** insecticides, photosynthetic rate, phytotoxicity, horticulture, pests and diseases, IPM

## Abstract

Although a large number of pesticides of different compositions are regularly used in agriculture, the impact of pesticides on the physiology of field crops is not well understood. Pesticides can produce negative effects on crop physiology―especially on photosynthesis―leading to a potential decrease in both the growth and the yield of crops. To investigate these potential effects in greenhouse sweet peppers, the effect of 20 insecticides and 2 fungicides (each sprayed with a wetting agent) on the photosynthesis of sweet pepper leaves was analyzed. Among these pesticides, nine caused significant reductions in photosynthetic activity. The effects were observed in distinctive ways—either as a transitory drop of the photosynthetic-rate values, which was observed at two hours after the treatment and was found to have recovered after 24 h, or as a sustained reduction of these values, which remained substantial over a number of days. The results of this study suggest that the production of a crop may substantially benefit when the frequent use of pesticides can be substituted with alternative pest control methods (e.g., biological control). Our results advocate further investigation of the potential impact of pesticides, either alone or in combination, on the photosynthesis of crop plants.

## 1. Introduction

After 60 years of wide-scale commercial use and despite the numerous negative consequences associated with pesticide treatments [1,2,3,4,5,6,7], pesticides still remain the quintessential strategy of crop management in most agricultural systems, with over 2.7 million metric tons used yearly, on a global scale [8]. The guidelines for the efficacy evaluation of pesticides of the European and Mediterranean Plant Protection Organization (EPPO) [9] describe the criteria for assessing phytotoxicity in host plants. These are the evident visual effects (necrosis, discoloration, and deformation) and the effects on yield (weight and appearance) after isolated treatments. Pesticides may impact the crop physiology through various disruptions, such as perturbation in the development of the reproductive organs, growth reduction, and alteration of the carbon and/or nitrogen metabolism, leading to a lower nutrient availability for plant growth. These disruptions will partly depend on the type of pesticide used [10]. Some important effects may only become apparent after repeated treatments and not necessarily translate into visible necrosis [11].

Different mechanisms have been described that explain the impact of pesticides on the photosynthesis process—the inhibition of the electron flow, the uncoupling of photophosphorylation, the solubilization of lipids, conformational changes [12,13], and/or mechanical effects [11]. Negative effects of applications of different pesticides on photosynthesis have been reported in a variety of crops, e.g., lettuce [14,15,16], cotton [17], alfalfa [18], citrus [19], strawberry [20], maize [21], peach [22], soybean [23], azalea [24], and pecan [25]. In a few studies, no significant reductions in photosynthesis were observed after exposure to insecticides [13,18,26]. In one study, there was even an increase [20].

Sweet pepper is, together with tomato, the most important horticultural greenhouse crop in Almeria (SE Spain), with its production covering a total surface area of approximately 10,000 ha (2019), generating a turnover of over 500 million euros [27,28]. The study presented in this paper was carried out between 2002 and 2004, when approximately 8,000 ha of sweet pepper greenhouses provided the main source of income for the province (COEXPHAL, unpublished data). The unprecedented concentration of greenhouses led to generalized pest problems, especially related to thrips (*Frankliniella occidentalis*) and the virus transmitted by this insect (Tomato Spotted Wilt Virus, TSWV). In most greenhouses, insecticides were applied weekly, usually with a mix of several phytosanitary products in each treatment [29]. As a result of this intensive insecticide regime, *F. occidentalis* developed extremely high levels of resistance to all available active ingredients [30], leading to a further increase in pesticide use.

The present study was initiated to quantify the short-term effect of pesticide treatments on the photosynthesis of sweet pepper leaves. It was performed in Almeria when Integrated Pest Management (IPM) adoption was in its initial stage [31]. Within this IPM system, biological control was directed at insect and mite pests, so the study was focused primarily on the effect of the most commonly used insecticides and acaricides. Since this study was carried out, two active ingredients―endosulfan and flufenoxuron―have been banned in the EU [32,33]. Nevertheless, both products are still frequently used in several other non-EU countries [34,35,36,37,38,39,40,41], so the results presented here are still relevant. Some of the other commercial products that were tested are no longer registered for their use in sweet pepper production in Spain. Additionally, the composition and/or commercial names of a number of products may have been subject to alterations.

## 2. Materials and Methods

A series of individual-leaf gas-exchange measurements were conducted in a 12 m^2^ growth room located at the Centre of Agricultural Research and Training (IFAPA). Altogether, 22 pesticides (20 insecticides and 2 fungicides) were tested (see Table 1 for details). The list of pesticides tested in this study was provided by the field advisors of a horticulture cooperative in Almeria (EUROSOL S.A.T.) and included the standard products used in the area for the protection of sweet pepper crops. As it was common practice among growers to add a wetting agent to each treatment, this was also done in this study.

Sweet pepper plants (*Capsicum annuum* cv. Vergasa, MN, USA) were grown in 13 cm diameter pots and kept in an isolated growth room to prevent the entrance of insect and mite pests. Throughout the study, the environmental conditions in the growth room were maintained between 12 and 15 °C and 23 and 27 °C, with 55%–75% relative humidity in the light/dark sequence, respectively. A photosynthetically active radiation (PAR) of 340 μmol m^−2^ s^−1^ at the top of the plant canopy was provided by fluorescent tubes for a daily photoperiod of 10 h. The growth room maintained a CO_2_ concentration of 375 ± 10 μmol mol^−1^. Coir (Fico^®^, imported by ISPEMAR S.C.A., Spain) was used as a substrate in the pots, and plants were fertilized using a standard nutrient solution. Plants were not sprayed or treated with any agrochemical before the gas-exchange measurements were taken. Around 6 weeks after being transplanted, the plants were ready for treatment measurements and were arranged with sufficient distance between them to avoid shading effects. Each plant was only used for one treatment on one of its leaves. Comparable, fully expanded (fourth or fifth leaf from the plant tip) and illuminated leaves of the same age were selected for the treatments. The application of the insecticide was conducted in a different room by the individual hand spraying of each of the selected leaves until dripping point. This method was found to be better than leaf dipping (i.e., the standard method in the evaluation of the efficiency of pesticides), because the latter did not provide a homogeneous distribution of insecticide solution droplets on the leaf. Light conditions in the treatment room were equal to those in the growth room.

Two treatments were carried out for each of the tested pesticides. A completely randomized experimental design was used, using nine replications per treatment. The leaves from the treated plants received a single application of one of the pesticides (at the recommended commercial dosage) dissolved in distilled water using a non-ionic wetting agent (nonylphenol polyethylene glycol ether 20% p/v SL; Commercial brand: Mojafel^®^; 100 cc hL^−1^). The leaves from the control (untreated) plants were sprayed with distilled water and received similar handling (control plants were transferred, like the treated ones, to the treatment room and back to the growth room).

A portable gas-exchange system (model Li-6400, Li-Cor, Lincoln, NE, USA) was used to measure the rate of photosynthesis in the sweet pepper leaves. Photosynthetic rates were determined from a 6 cm^2^ leaf area measured by the leaf chamber of the Li-6400, which included an internal light source adjusted to the Photosynthetically Active Radiation (PAR) conditions in the growth room. Photosynthesis was measured on three occasions before treatment (48, 24, and 2 h before) and six occasions after treatment (2, 24, 48, 72, 96, and 120 h afterward). All measurements were taken between 09:00 and 12:00. The measurements were stopped after a week, as, due to the growth of the plants, the treated leaves were partly shaded by new leaves, possibly altering the photosynthesis pattern.

Data were analyzed using the ANOVA test, and separation of means at *p* < 0.05 was analyzed by Fisher’s least significance difference procedure. Before the analysis, data were checked and transformed when necessary to ensure normality and homoscedasticity. Data analyses were performed with Statgraphics software.

## 3. Results

Nine out of the 22 studied pesticides produced a significant decrease in the photosynthesis rates of sweet pepper leaves after a single application. However, the impact on the photosynthesis rate, as well as the moment and duration of the impact, varied between pesticides (Table 1).

**Figure 1:**
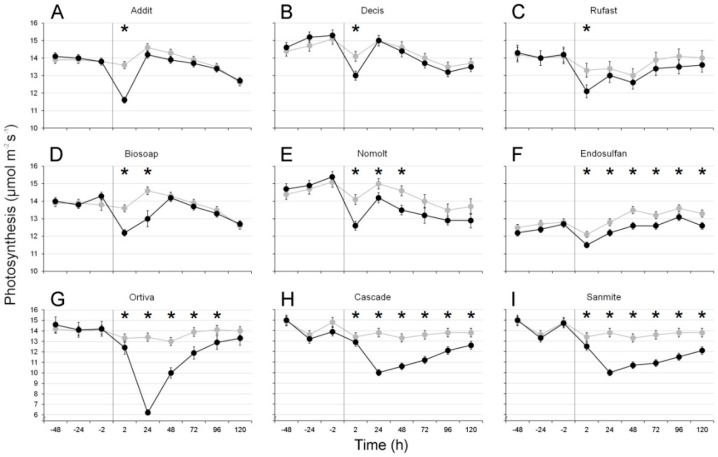
Mean ± SE photosynthetic rates (μmoL CO_2_ m^−2^ s^−1^) of sweet pepper leaves before and after treatment with nine different pesticides. Gray curves refer to control plants; black curves indicate treated plants. The moment when each treatment was conducted is indicated by a vertical line (i.e., time = 0). * Indicates significant differences (*p* < 0.05) between treatments at a given time.

Treatments with five products caused instantaneous reductions in photosynthesis, with a maximum in the measurement being reached at 2 h post treatment. For three of these―Addit, Decis, and Rufast―this effect was transient, as no more significant differences were observed after the first measurement (Table 1, Figure 1A–C). Treatment with Biosoap led to significant reductions until at least 24 h after treatment, whereas those produced by Nomolt lasted 48 h (Table 1, Figure 1D,E).

Three products―Ortiva, Cascade, and Sanmite―caused a maximum photosynthesis reduction after 24 h. In these cases, the effects were substantial, with maximum reductions of 54%, 28%, and 28%, respectively (Table 1, Figure 1G–I), and the values recovered 96 h after the treatment (Ortiva) or had not recovered after 5 days (Cascade and Sanmite). The leaves sprayed with endosulfan showed significant, consistent reductions of the photosynthesis rate (between 4% and 7%) from two hours after the treatment until the last measurement was taken (i.e., 120 h after treatment) (Table 1, Figure 1F).

## 4. Discussion

This study showed significant effects on the photosynthesis of 9 out of 22 investigated phytosanitary products. Two types of effects can be distinguished. The first is a transient effect, immediately after the treatment, possibly caused by a short-term effect on the stomatal conductance. The group of phytosanitary products that has this type of effect includes a vegetable oil (Addit) and an organic soap (Biosoap). Similar compounds are frequently used as wetting agents, surfactants, etc. in phytosanitary products, so the effect of the formulated products might sometimes be explained by the adjuvants rather than by the effect of the active ingredients. Insecticides and surfactants with oil-based formulations can produce more severe negative effects on crop gas exchange in comparison with other formulations [11]. The second type of effect is a maximum reduction after 24 h or more, indicating a somewhat slower chemical interaction between the pesticide residues and the leaf tissue. This effect can persist, as observed with Ortiva, Cascade, and Sanmite. Although it does not lead to visible phytotoxic symptoms in the shape of necrotic spots, it is obvious that this reduction may have a significant effect on plant growth and production. Photosynthetic-rate reductions could have been partly produced by the addition of the wetting agent (i.e., Mojafel ^®^). Although 13 out of the 22 treatments, which contained the wetting agent, did not alter the photosynthetic-rate results, a potential interaction between the wetting agent and certain pesticides cannot be ruled out. Under commercial greenhouse conditions, the reduction in plant growth caused by treatments is likely to be more important than the effects observed in this study. Growers usually spray with a mix of various phytosanitary products in the same solution. Possible synergies in the effects of these products should be investigated in further detail. Furthermore, in insecticide-controlled crops, the frequency of treatments may be weekly for most of the year, causing accumulative effects that have, thus far, not been quantified. A not-well-documented but clearly visible effect of frequent pesticide treatments, which could not be addressed in this study, is a gradual physical distortion of the leaves that causes the older leaves to curl up, reducing the exposed surface for photosynthesis.

Since, as found in this study, the effects of pesticides on plant physiology can be substantial and due to the fact that there are marked differences between the effects of different products, it would be helpful to document the direct effects of treatments on photosynthesis for all registered products. This could help growers and technicians in their decision making, e.g., in cost-benefit calculations when they have to choose between alternative pest control strategies.

Potential adverse effects of pesticide treatments can be avoided by adopting alternative pest control techniques. Especially in greenhouse horticulture, biological control, through arthropod predators and parasitoids, can largely replace the use of chemical insecticides [42]. In Almeria, IPM, using biological control as a fundamental component, was implemented on a large scale in 2007 [29]. An important reason for this massive change was that growers observed IPM crops with better plant growth and a higher productivity than that of crops grown in greenhouses with continuous insecticide treatments (COEXPHAL, unpublished data).

## Figures and Tables

**Table 1:** List of the studied pesticides (20 insecticides and 2 fungicides (in italics)), and their effects on the photosynthesis rates of sweet pepper leaves.

Commercial Name	Active Ingredient	Cc	Maximum Reduction (%)	Moment (h)	Duration (h)
Addit^®^	Plant oil	250 cc/hL	15%	2	2
Align^®^ (3.2% p/v EC)	Azadirachtin	150 cc/hL	NS	-	-
Apache^®^ (1.8% EC)	Abamectin	60 cc/hL	NS	-	-
Applaud^®^ (25% WP)	Buprofezin	80 gr/hL	NS	-	-
Biosoap^®^	Fatty acids	1000 cc/hL	11%	2	24
Cascade^®^ (10% p/v DC)	Flufenoxuron	100 cc/hL	28%	24	120
Confidor^®^ (20% p/v SL)	Imidacloprid	75 cc/hL	NS	-	-
Decis^®^ (2.5% p/v EC)	Deltamethrin	50 cc/hL	8%	2	2
Dicarzol^®^ (50% SP)	Formetanate	200 gr/hL	NS	-	-
Endosulfan (35% p/v EC)	Endosulfan	300 cc/hL	7%	48	120
*Galben* ^®^ *(8% + 65% WP)*	*Benalaxyl + Mancozeb*	*250 gr/hL*	*NS*	*-*	*-*
Karate King^®^ (2.5% WG)	Lambda-cyhalothrin	80 gr/hL	NS	-	-
Malafin 90^®^ (90% p/v EC)	Malathion	200 cc/hL	NS	-	-
Match^®^ (5% p/v EC)	Lufenuron	200 cc/hL	NS	-	-
Mimic^®^ (24% p/v SC)	Tebufenozide	75 cc/hL	NS	-	-
Nomolt^®^ (15% p/v SC)	Teflubenzuron	60 cc/hL	11%	2	48
*Ortiva* ^®^ *(25% p/v SC)*	*Azoxystrobin*	*80 cc/hL*	*54%*	*24*	*96*
Ripcord^®^ (10% p/v EC)	Cypermethrin	100 cc/hL	NS	-	-
Rufast^®^ (7.5% EW)	Acrinathrin	80 cc/hL	9%	2	2
Sanmite^®^ (20% WP)	Pyridaben	100 gr/hL	28%	24	120
Sulfur (80% WP)	Wettable sulfur	400 gr/hL	NS	-	-
Trigard^®^ (75% WP)	Cyromazine	40 gr/hL	NS	-	-

Maximum reduction indicates the reduced percentage (compared with control plants) of the photosynthetic rate due to pesticide application at the recommended concentrations (Cc), moment of maximum reduction indicates the time (h) at which the maximum reduction occurred, and duration of the effect indicates the period of time (h) during which significant differences in the photosynthesis rate between the control and treated plants occurred. EC: emulsifiable concentrate; EW: emulsion, oil in water; SC: suspension concentrate; WP: wettable powder; DC: dispersible concentrate; SL: soluble concentrate; WG: water dispersible granule; NS: no significant effect observed.

## References

[1] Roditakis E., Vasakis E., García-Vidal L., del Rosario Martínez-Aguirre M., Rison J.L., Haxaire-Lutun M.O., Nauen R., Tsagkarakou A., Bielza P. (2018). A four-year survey on insecticide resistance and likelihood of chemical control failure for tomato leaf miner *Tuta absoluta* in the European/Asian region. J. Pest Sci..

[2] Chintalapati P., Katti G., Puskur R.R., Nagella Venkata K. (2016). Neonicotinoid-induced resurgence of rice leaffolder, *Cnaphalocrocis medinalis* (Guénee). Pest Manag. Sci..

[3] Desneux N., Decourtye A., Delpuech J.M. (2007). The sublethal effects of pesticides on beneficial arthropods. Annu. Rev. Entomol..

[4] Mac Loughlin T.M., Peluso L., Marino D.J. (2017). Pesticide impact study in the peri-urban horticultural area of Gran La Plata, Argentina. Sci. Total Environ..

[5] Hansen B., Alrøe H.F., Kristensen E.S. (2001). Approaches to assess the environmental impact of organic farming with particular regard to Denmark. Agric. Ecosyst. Environ..

[6] Alavanja M.C. (2009). Introduction: Pesticides use and exposure, extensive worldwide. Rev. Environ. Health.

[7] Gomiero T., Pimentel D., Paoletti M.G. (2011). Environmental impact of different agricultural management practices: Conventional vs. organic agriculture. Crit. Rev. Plant Sci..

[8] Environmental Protection Agency (EPA) Website Pesticides Industry Sales and Usage. https://www.epa.gov/sites/production/files/2017-01/documents/pesticides-industry-sales-usage-2016_0.pdf.

[9] European and Mediterranean Plant Protection Organization (EPPO) (2014). Efficacy evaluation of plant protection products, PP 1/135 (4) Phytotoxicity assessment. Bull. OEPP/EPPO Bull..

[10] Petit A.N., Fontaine F., Vatsa P., Clément C., Vaillant-Gaveau N. (2012). Fungicide impacts on photosynthesis in crop plants. Photosynth. Res..

[11] Ferree D.C., Marcelle R., Clijsters H., van Poucke M. (1979). Influence of pesticides on photosynthesis of crop plants. Photosynthesis and Plant Development.

[12] Murthy C.S.H.N. (1983). Effect of pesticides on photosynthesis. Residue Rev..

[13] Krugh B.W., Miles D. (1996). Monitoring the effects of five “nonherbicidal pesticide chemicals on terrestrial plants using chlorophyll fluorescence”. Environ. Toxicol. Chem..

[14] Toscano N.C., Sances M.W., Jonson M.W., LaPré L.F. (1982). Effect of various pesticides on lettuce physiology and yield. J. Econ. Entomol..

[15] Johnson M.W., Welter S.C., Toscano N.C., Iwata Y., Ting P. (1983). Lettuce yield reductions correlated with methyl parathion use. J. Econ. Entomol..

[16] Haile F.J., Kerns D.L., Richardson J.M., Higley L.G. (2000). Impact of insecticides and surfactant on lettuce physiology and yield. J. Econ. Entomol..

[17] Youngman R.R., Leigh T.F., Kerby T.A., Toscano N.C., Jackson C.E. (1990). Pesticides and cotton: Effect on photosynthesis, growth, and fruiting. J. Econ. Entomol..

[18] Haile F.J., Peterson R.K.D., Higley L.G. (1999). Gas-exchange responses of alfalfa and soybean treated with insecticides. J. Econ. Entomol..

[19] Jones V.P., Youngman R.R., Parrella M.P. (1983). Effect of selected acaricides on photosynthetic rates of lemon and orange leaves in California. J. Econ. Entomol..

[20] Laprè L.F., Sances F.V., Toscano N.C., Oatman E.R., Voth V., Johnson M.W. (1982). The effects of acaricides on the physiology, growth, and yield of strawberries. J. Econ. Entomol..

[21] Godfrey L.D., Holtzer T.O. (1992). Effects of soil-incorporated insecticides and foliar applied chemicals on corn gas-exchange parameters. Crop Prot..

[22] Andersen P.C., Mizell R.F., French W.J., Aldrich J.H. (1986). Effect of multiple applications of pesticides on leaf gas exchange of peach. Hort. Sci..

[23] Abdel-Reheem S., Belal M.H., Gupta G. (1991). Photosynthesis inhibition of soybean leaves by insecticides. Environ. Pollut..

[24] Kingleman W.E., Buntin G.D., van Iersel M.W., Braman S.K. (2000). Whole plant gas exchange, not individual leaf-measurements, accurately assesses azalea response to insecticides. Crop Prot..

[25] Wood B.W., Payne J.A. (1986). Net photosynthesis of orchard grown pecan leaves reduces by insecticide sprays. Hort. Sci..

[26] Lloyd R.W., Krieg D.R. (1987). Cotton development and yield as affected by insecticides. J. Econ. Entomol..

[27] Cajamar Website Análisis de la Campaña Hortofrutícola de Almería, Campaña 2017/2018. https://www.publicacionescajamar.es/series-tematicas/informes-coyuntura-analisis-de-campana/analisis-de-la-campana-hortofruticola-de-almeria-campana-20172018.

[28] Junta de Andalucía Website Avance de Superficies y Producciones. https://www.juntadeandalucia.es/organismos/agriculturaganaderiapescaydesarrollosostenible/servicios/estadisticas/detalle/69831.html.

[29] Van der Blom J. (2009). Microbiological insecticides against lepidopteran pests in greenhouse horticulture in Almeria, Spain. Bull. OEPP/EPPO Bull..

[30] Bielza P. (2008). Insecticide resistance management strategies against the western flower thrips, *Frankliniella occidentalis*. Pest Manag. Sci..

[31] Van der Blom J. (2010). Applied entomology in Spanish greenhouse horticulture. Proc. Neth Entomol. Soc. Meet..

[32] Pesticide Properties Database Website Endosulfan. https://sitem.herts.ac.uk/aeru/ppdb/en/Reports/264.htm.

[33] Pesticide Properties Database Website: Flufenoxuron. https://sitem.herts.ac.uk/aeru/ppdb/en/Reports/332.htm.

[34] Menezes R.G., Qadir T.F., Moin A., Fatima H., Hussain S.A., Madadin M., Senthilkumaran S. (2017). Endosulfan poisoning: An overview. J. Forensic Leg. Med..

[35] Isogai N., Hogarh J.N., Seike N., Kobara Y., Oyediran F., Wirmvem M.J., Ayonghe S.N., Masunaga S. (2016). Atmospheric monitoring of organochlorine pesticides across some West African countries. Environ. Sci. Pollut. Res..

[36] Ibrahim E.G., Yakubu N., Nnamonu L., Yakubu J.M. (2018). Determination of organochlorine pesticide residues in pumpkin, spinach and sorrel leaves grown in Akwanga, Nasarawa State, Nigeria. J. Environ. Prot..

[37] Kumar M., Philip L., Singh S.N. (2017). Remediation of endosulfan contaminated system by microbes. Microbe-Induced Degradation of Pesticides.

[38] Radhakrishnan S. (2018). A note on wildlife poisoning cases from Kerala, South India. Eur. J. Wildlife Res..

[39] Shaurub E.S.H., Zohdy N.Z., Abdel-Aal A.E., Emara S.A. (2018). Effect of chlorfluazuron and flufenoxuron on development and reproductive performance of the black cutworm, *Agrotis ipsilon* (Hufnagel)(Lepidoptera: Noctuidae). Invertebr. Reprod. Dev..

[40] Chang J., Li W., Guo B., Xu P., Wang Y., Li J., Wang H. (2017). Unraveling the different toxic effect of flufenoxuron on the thyroid endocrine system of the *Mongolia racerunner* (Eremias Argus) at different stages. Chemosphere.

[41] Suzuki Y., Shiotsuki T., Jouraku A., Miura K., Minakuchi C. (2017). Benzoylurea resistance in western flower thrips *Frankliniella occidentalis* (Thysanoptera: Thripidae): The presence of a point mutation in chitin synthase 1. J. Pestic. Sci..

[42] Van Lenteren J.C., Charles Vincent C., Goettel M.S., Lazarovits G. (2007). Biological control for insect pests in greenhouses: An unexpected success. Biological Control: A Global Perspective: Case Studies from Around the World.

